# Exploring the Impact of Group Size on Medical Students’ Perception of Learning and Professional Development During Clinical Rotations

**Published:** 2018-08-30

**Authors:** Samuel Ofei-Dodoo, Kyle Goerl, Scott Moser

**Affiliations:** University of Kansas School of Medicine-Wichita, Department of Family and Community Medicine

**Keywords:** undergraduate medical education, clinical clerkships, group structure, professional autonomy

## Abstract

**Introduction:**

Research assessing the size of learning groups in medical education and how that affects the learner’s experience is limited. The main goals of the study were to (1) assess the effect of varying group size on medical students’ subjective experiences during clinical years. We hypothesized that students in smaller groups were more likely to have better experiences during clinical rotation than those in larger groups, and (2) determine if medical students have desirable experiences working with other medical learners (fellows, residents, osteopathic students, physician assistants, and nurse practitioners) during clinical rotations.

**Methods:**

The study utilized a mixed method approach where 153 medical students in their clinical years were asked to complete a 10-item survey. A linear-by-linear association test of trend and Mann-Whitney U test were used to evaluate the students’ quantitative data. A multidisciplinary team used an immersion-crystallization approach to analyze the content of the students’ qualitative data.

**Results:**

There was a 90% (137/153) response rate. Most students (80%) reported desirable experiences during clinical rotations because of supportive learning environments, engaging preceptors, willingness of residents to teach, as well as the opportunity to participate in patient care. There were significant differences in students’ perceived clinical experiences as a function of group size, where groups of two students were preferable over groups of four or more.

**Conclusions:**

Varying group size appears to affect students’ clinical experiences.

## INTRODUCTION

Clinical rotations play a vital role in medical education. Clinical rotations give clinical learners (fellows, residents, medical students, physician assistants, and nurse practitioner students) exposure to a wide variety of specialties, while interacting with higher-level learners and attending physicians. Group educational experiences vary within clinical rotations with opportunities including bedside rounds, self-study, didactics, and problem-based learning.

Learner’s experience improves with the quality of the educator’s communication skills and clinical expertise.^1^ Those factors also can play a role in a learner’s career choice. Clinical learners rate clinical rotations higher when they are well organized, well supervised, the learner is integrated fully into the experience, and there is opportunity to improve clinical skills.^1^,^2^

In contrast, literature from nursing education shows students placed in “unhealthy” environments that consist of lack of respect, trust, and support are likely to have increased psychosocial risks that often lead to reduced satisfaction in their clinical experiences.^3^ Additionally, despite evidence showing that learners appreciate and prefer learning by way of bedside rounds, this activity can be limited when the number of learners in a group is large.^4^ Interestingly, learners are not the only ones who benefit from bedside rounds, as the patient experience improves as well.^5^ The patient perceives that more compassionate care is provided in this setting.

To provide learners with ample patient exposure, bedside teaching can include a large number of learners. Understandably, medical students could feel lost in a large group and may experience a lack of support or even disrespect if attention is not directed toward them. Conceivably, this could happen even when the attending is doing his or her best to create a healthy learning environment.

Logically, most attending physicians want to provide adequate oversight of their learners in addition to trying to make them feel like they are an important part of the team. At a basic level, accessibility to the attending is important to ensure the students feel supported; however, the availability of an attending to students can be misperceived. Physicians believe they are more accessible to their students than the learners think they are.^6^ If the attending feels like they are available, but the student does not, this could create an environment where the student feels neglected and be the reason why twice as many students think that first and second year residents are better teachers than physicians.^6^

There is very little research assessing the size of clinical learning groups in medical education and how that affects the learner’s experience. Rezmer and colleagues^7^ have demonstrated there is no difference in the medical students’ experiences during a resuscitation simulation learning module with groups ranging in size from two, three, or four. The current study seeks to find if there is a difference in the medical students’ clinical experience based on learner group size. For the purposes of this study, we defined “clinical experience” as students’ perception of clinical proficiency, professional development, and access to attending during clinical rotations. Specifically, the study seeks to gather more information related to the following questions:

1. Are there differences among medical students’ experiences working in groups ranging from two, three, or four? We hypothesized that medical students (MS) in larger groups were less likely to have desirable clinical experiences than students in smaller groups.
2. Does group size of clinical learners (fellows, residents, osteopathic students, physician assistants, and nurse practitioners) affect medical students’ perception of clinical proficiency, professional development, and access to attending during clinical rotations?
3. Do medical students have better clinical experiences when working with other clinical learners? If so, why?

## METHODS

### Study Design

This non-experimental, cross-sectional study relied on third (MS3) and fourth year medical students (MS4) to complete a survey to assess their experiences working with other clinical learners during clinical rotations. The convenience sample of third and fourth year medical students was used because the students were in their clinical years at a local medical school. The study utilized a mixed method approach^8^,^9^ (integrating both qualitative and quantitative questions) to collect, analyze, and interpret the data. The use of qualitative design, specifically, provided the researchers with an opportunity to develop an in-depth understanding into factors that improve students’ clinical experiences. The University of Kansas School of Medicine-Wichita IRB granted an exemption for the study.

### Study Instrument

The data used for this study are part of a larger study that examined MS perception of clinical proficiency, professional development, and comfortability working with other clinical learners during clinical years. During our literature search, we were not able to find a previously validated survey instrument that met our needs. Therefore, we developed a 10-item survey questionnaire (see Appendix A) to measure the participants’ perception of working in groups during clinical rotations. First, the items were created based on the goal of the study. The generated questions were reviewed by the local associate dean for medical student curriculum and the director of the family medicine clerkship, who have experience in MS clinical clerkship development and implementation, to ensure that the questions accurately assessed the constructs identified in the study. A group of MS3s vetted the questionnaire to ensure that the items had face validity. The students who vetted the questionnaire did not participate in the actual study. Medical students in their clinical years (N = 153) were requested to complete the survey between December 2016 and May 2017. The authors used a paper-and-pencil approach to collect the data.

### Statistical Analyses

A linear-by-linear association test of trend was calculated to determine if the students’ experiences with clinical rotations related to their group size. Follow-up pairwise comparisons were conducted to evaluate differences among the proportions from the association test. The Mann-Whitney U test also was conducted to evaluate whether students’ perception of clinical proficiency, professional development, and access to attending differed based on their clinical experiences during rotations. A *priori* analysis was used to power the study based on asymptotic relative efficiency adjustment of 0.988 for nonparametric Mann Whitney U test.^8^ Assuming a criterion value of α = .05, *d* = .05, adjusted N = 144 (137/0.988), the priori statistical power was 0.98.

A multidisciplinary team utilized an immersion-crystallization approach^9^–^12^ to analyze the qualitative data. The team comprised of a community psychologist (SO-D), a family physician (KG), and a family physician and an associate dean for curriculum (SM). An immersion-crystallization is a dual process where researchers examine collected data in details, momentarily suspend the immersion process to reflect on the data, and attempt to identify observed themes during the immersion process.^9^–^11^

## RESULTS

### Quantitative Results

Of the 153 total medical students in the third and fourth year classes on a local campus, data were collected from 137, an 89.5% response rate. Of the 135 respondents who provided their sex, 54.1% were men and 45.9% were women. The majority of the respondents (77.4%) were Caucasian (Table 1). Eighty percent (110) of the students reported that they had desirable experiences during their clinical rotations and provided several reasons (these are discussed later, under the qualitative results section). The linear-by-linear association test showed a statistical association between the students’ clinical experiences and group size (χ^2^ (134) = 6.1, *p* = .014; Table 2). The proportion of students with group size of two, three, or four who had desirable clinical experiences was .87, .70, and .67 respectively.

Follow-up pairwise comparisons were conducted to evaluate the differences among the proportions (Table 3) with the Holm sequential Bonferroni method to control for Type I error at the 0.05 level across all three comparisons. The only significant pairwise difference was between group sizes of two and four. The probability of a student having desirable clinical experience was 1.30 times (.87/.67) more likely when s/he was in a group size of two learners as opposed to group size of four.

As shown in Table 4, working in groups did not have an effect on students’ perceptions of their clinical proficiency, professional development, and access to attending physicians at the end of the rotation. However, when median scores were analyzed as a function of students’ level of clinical experiences, students who reported undesirable experiences felt working with other clinical learners (1) interfered with their clinical proficiency (*z* = −3.77, *p* < .001; median of desirable = 3.0, median of undesirable = 2.0), and (2) negatively affected their access to the attending (*z* = −3.99, *p* < .001; median of desirable = 2.0, median of undesirable = 1.0).

### Qualitative Results

#### Medical students who had desirable clinical experiences

Of the 137 respondents, 110 (80%) reported to have had desirable clinical experiences during their clinical rotations. Four interconnected themes emerged from their data analyses as reasons for better clinical rotations: safe and supportive learning environments, hands-on experiences, engaging preceptors, and willingness of residents to help. Each of these themes is discussed in detail in the subsequent sections.

#### Safe and supportive learning environment

Of the 110 students who reported to have had better clinical experiences, 38 (35%) credited supportive learning environments. MS A stated “great working environment and lots of support from fellow students and residents.” This sentiment was shared by MS B who explained “supportive learning environment, and flexibility to learn in different settings were very helpful.” Other students felt that working with other clinical learners improved their clinical experiences because of shared responsibilities. MS C wrote “having other learners there provided additional point of view to learn from.” Likewise, working in an environment where students felt comfortable to ask questions improved clinical experiences. MS D explained “I felt comfortable asking questions, safe learning environment.” MS E’s response typified a safe learning environment where students can ask questions: “I was able to ask questions and fully participate.”

#### Hands-on experiences

Thirty-two percent (35 of 110) of the participants indicated that they had desirable clinical experiences because they were involved in patient care during their clinical rotation. MS F explained “it was very hands-on, and I learned a lot.” This sentiment was echoed by MS G: “lots of hands-on experiences.” Some participants also indicated that working one-on-one with preceptors was a great experience. MS H wrote “I worked with a physician one-on-one and got a great experience.”

#### Engaging preceptors

Nineteen percent (21 of 110) of the participants who had desirable clinical experiences expressed that their interaction with the attending physicians made their experiences worthwhile. Specifically, it was the eagerness of the attending to educate the students that helped the latter enjoy their clinical experiences. MS I wrote “the attending was very good at teaching and engaging all the students and residents.” MS J also made a similar observation: “attending took time to teach as well as discuss cases and answer questions.”

#### Willingness of residents to help

Fourteen percent (15 of 110) of the students explained that they had good clinical experiences because of supportive residents who were willing to help. MS K stated “I had residents who were willing to teach and guide my thinking.” MS L wrote “residents were extremely helpful in directing us to information we would need to know to benefit our patients.”

#### Medical students who had undesirable clinical experiences

Twenty percent (27 of 137) of the respondents reported undesirable experiences with their clinical rotations. Three interconnected themes emerged from their data analyses as reasons for having undesirable clinical rotations: not enough patient exposure, group size/composition, and not enough one-on-one with attending.

#### Not enough patients to see

Thirty-seven percent (10 of 27) of the students attributed their undesirable clinical experiences to not having enough patient exposure for meaningful experiences. MS M who stated “There were not many patients to see during the rotations.”

#### Group size and composition

Thirty-seven percent (10 of 27) of the students indicated that their undesirable clinical experiences were due to the group size of clinical learners working with a preceptor in busy clinical practices. MS N stated “I didn’t get the most out of my summer rotation as there were about 4 learners working with a preceptor in a fast-paced environment.” Likewise, other students did not like working in groups because of group dynamics. MS O wrote “grouping us together creates cues for group conformity that puts the impetus on not speaking. [It] was more difficult to ask questions.” A similar observation was shared by MS P who indicated “too many learners reinforces the traditional hierarchical way of learning that is less effective and efficient than personalized, mutual respect learning.”

#### Not enough time with attending

Twenty-six percent (7 of 27) of the students attributed their undesirable clinical experiences to not having enough time with the attending physicians. MS Q explained “not enough time with the attending and senior residents during the first four weeks.” MS R’s statement typified how the preceptors’ workloads affected students’ clinical experiences: “Residents and attending were too busy to teach.”

## DISCUSSION

This study provided information regarding medical students’ experiences during clinical years of medical education at our institution. The findings demonstrated that most students have positive experiences during clinical years. Our data suggested that to provide optimal and quality learning experiences to medical students, they should be placed in supportive learning environments where they can participate in patient care confidently under the supervision of engaging preceptors and helpful residents.

The major findings demonstrated the impact of group size on students’ clinical experiences, clinical proficiency, professional development, and access to attending physician. In particular, group size was a significant predictor for students’ satisfaction during clinical experiences. Unlike the study by Rezmer and colleagues^7^ who demonstrated no effect of group size on medical students’ experiences during a resuscitation simulation learning module, our findings in clinical environments have shown there are differences among students’ experiences working in groups ranging from two, three, or four in clinical settings.

Working in groups with other clinical learners provides camaraderie as learners can rely on each other for ideas and support, but group size and composition can affect students’ learning experiences negatively. An advantage of working in smaller groups included students having more patient contact time, more contact time with attending and senior residents, and confidence in asking questions. Students in larger groups were less likely to ask questions and/or share an opinion on cases, especially in fast-paced clinical environments where medical teams have little time to care for many patients. One could argue that a group composed of learners at various levels of medical education would enhance knowledge, as clinical learners are able to learn from each other. However, larger groups often result in the traditional hierarchical way of learning where clinical attention is based on seniority. Thus, working with others in large groups could interfere with students’ learning as well as negatively affect their access to the attending. Further research is indicated into why group size influenced medical students’ perception of clinical proficiency development and access to the attending physicians, but did not have any effect on professional development.

The study had several limitations including a small sample size limiting generalizability of the findings, but it provided data on how group size affects medical students’ experiences during clinical rotations. The study also was limited in its diversity. It was conducted at a single, urban medical educational institution and majority of its participants were Caucasian. Future studies should include larger and more diverse samples of students from other medical schools. Self-reported clinical experiences and possible recall bias also limit the findings of the study. Additionally, the study was limited by the fact that it measured medical student perception and not the impact on clinical opportunities during rotations.

In conclusion, our study has drawn attention to the evaluation of students’ clinical experiences in medical education. To our knowledge, this is the first assessment that has looked into the effect of group size on medical students’ experience in clinical settings. Varying group size appears to have an effect on medical students’ clinical experiences. Medical students are more likely to have desirable clinical experiences when they are in smaller groups.

## Figures and Tables

**Table 1:** Demographic profile of participants (n = 137).

Demographic of Participants	Measure
Sex, *no.* (%)	
Male	73 (54.1)
Female	62 (45.9)
Missing	2
Age	
Mean (SD)	27.6 (2.4)
Range	25 to 38 years
Missing	2
Ethnicity, *no.* (%)	
African American	6 (5.4)
Caucasian	103 (77.4)
Hispanic/Latino	7 (5.3)
Asian	10 (5.7)
Bi-racial	5 (3.8)
Other	2 (1.5)
Missing	4
Year in medical school, *no.* (%)	
MS 3	73 (53.3)
MS 4	64 (46.7)

**Table 2:** Results of linear-by-linear test and descriptive statistics for medical students working in groups.

	Clinical Experience		
Group Size	Desirable	Undesirable	Total
Group size = 2			
Count	73	11	84
Expected count	67.2	16.8	84.0
Percentage within group size	86.9	13.1	100.0
Group size = 3			
Count	21	9	30
Expected count	24.0	6.0	30.0
Percentage within group size	70.0	30.0	100.0
Group size ≥ 4			
Count	14	7	21
Expected count	16.8	4.2^a^	21.0
Percentage within group size	66.7	33.3	100.0
Total			
Count	108	27	135
Expected count	108.0	27.0	135.0
Percentage within group size	80.0	20.0	100.0

Note: χ^2^ = 6.1, *p* = 0.14; *df* = 1

a Has expected count of less than 5.

The minimum expected count is 4.20.

**Table 3:** Results for the pairwise comparisons using the Holm’s Sequential Bonferroni method.

Comparison	Pearson Chi-Square	*p* value (Alpha)	Cramer’s *V*
Group size = 2 vs. Group size = 4	4.80*	.028 (0.50)	0.22
Group size = 2 vs. Group size = 3	4.33	0.37 (0.25)	0.20
Group size = 3 vs. Group size = 4	0.06	.80 (.017)	0.35

* *p* value ≤ alpha

**Table 4:** Descriptive statistics and Mann-Whitney U test on clinical experiences in terms of professional development and access to attending physicians.

					Clinical Experiences					
	Overall Descriptive Statistics				Desirable		Undesirable			
	Score	n	%	Median (IQR)	n	Median (IQR)	n	Median (IQR)	*z*	*p* value
**Clinical Proficiency (N = 137)**				**3.0 (3.0 – 4.0)**	**108**	**3.0 (3.0 – 4.0)**	**27**	**2.0 (2.0 – 4.0)**	**−3.77**	**0.0001**
Having more than one medical learners working with the same educator...										
greatly interfered with my learning or clinical proficiency	1	3	2.2							
interfered with my learning or clinical proficiency	2	27	20							
did not have any effect	3	47	34							
improved my learning or clinical proficiency	4	53	39							
greatly improved my learning or clinical proficiency	5	7	5.1							
**Professional Development (N = 137)**				**3.0 (3.0 – 4.0)**	**108**	**3.0 (3.0 – 4.0)**	**27**	**3.0 (2.0 – 3.0)**		**0.0001**
Having more than one medical learners working with the same educator...										
greatly interfered with my professional development	1	2	1.5							
interfered with my professional development	2	17	12.4							
did not have any effect	3	63	46.0							
improved my professional development	4	45	32.8							
greatly improved my professional development	5	10	7.3							
**Access to the Educator (N = 137)**				**2.0 (1.0 – 2.0)**	**108**	**2.0 (2.0 – 2.0)**	**27**	**1.0 (1.0 – 2.0)**		**0.0001**
The number of medical learners working with an educator...										
negatively affected my access to the educator	1	37	27.0							
did not affect my access to the educator	2	79	57.7							
positively affected my access to the educator	3	21	15.3							

IQR = Interquartile range

## APPENDIX A Medical Students’ Perception of Experiences Survey

This questionnaire is part of a study to find out about your experiences working with other medical learners (residents, medical students, osteopathic (DO) student, physician assistant students, and nurse practitioner students) during your previous clinical rotations. The survey will take approximately five minutes to complete. Your participation is voluntary, and your responses will remain confidential. Your decision to participate will not in any way affect your standing at KUSM - Wichita now or in the future. We greatly appreciate your feedback. If you have any questions, please contact Dr. Samuel Ofei-Dodoo at sofeidodoo@kumc.edu or Dr. Kyle Goerl at Kyle.Goerl@via-christi.org.

1. Having more than one medical learner working with the same educator/supervisor…
  1. Greatly interfered with my learning or clinical proficiency
  2. Interfered with my learning or clinical proficiency
  3. Did not have any effect
  4. Improved my learning or clinical proficiency
  5. Greatly improved my learning or clinical proficiency
2. Having more than one medical learner working with the same educator/supervisor…
  1. Greatly interfered with my professional development
  2. Interfered with my professional development
  3. Did not have any effect
  4. Improved my professional development
  5. Greatly improved my professional development
3. The number of medical learners working with an educator/supervisor…
  1. Negatively affected my access to the educator/supervisor
  2. Did not affect my access to the educator/supervisor
  3. Positively affected my access to the educator/supervisor
4. During your previous clinical rotation, on average, how many learners (yourself included) worked with the same educator/supervisor at one time?
  1. 2
  2. 3
  3. 4
  4. Other (Please specify) _________________
5. Did you get the most out of your clinical experiences during your previous rotation? ___Yes ___No
  - Why and why not? ______________________________________________________________________________________________________________________________________________________________________________________________________________________________________________________________________________
6. In your opinion, what should be done to improve clinical rotations so you could get the most out of the experience? ____________________________________________________________________________________________________________________________________________________________________________________________________________________________________________________________________
  Please tell us about yourself:
7. Year of birth _________
8. Gender: ______Male ______Female
9. Race or cultural group do you identify with? _(please circle one)_
  1 = African American
  2 = Caucasian
  3 = Hispanic/Latino
  4 = Asian
  5 = Bi-racial
  6 = Other
10. I am a…
  1 = third year medical student
  2 = fourth year medical student
